# Family-specific genotype arrays increase the accuracy of pedigree-based imputation at very low marker densities

**DOI:** 10.1186/s12711-019-0478-2

**Published:** 2019-06-26

**Authors:** Andrew Whalen, Gregor Gorjanc, John M. Hickey

**Affiliations:** The Roslin Institute and Royal (Dick) School of Veterinary Studies, The University of Edinburgh, Midlothian, Scotland UK

## Abstract

**Background:**

In this paper, we evaluate the performance of using family-specific low-density genotype arrays to increase the accuracy of pedigree-based imputation. Genotype imputation is a widely used tool that decreases the costs of genotyping a population by genotyping the majority of individuals on a low-density array and using statistical regularities between the low-density and high-density individuals to fill in the missing genotypes. Previous work on population-based imputation has found that it is possible to increase the accuracy of imputation by maximizing the number of informative markers on an array. In the context of pedigree-based imputation, where the informativeness of a marker depends only on the genotypes of an individual’s parents, it may be beneficial to select the markers on each low-density array on a family-by-family basis.

**Results:**

In this paper, we examined four family-specific low-density marker selection strategies and evaluated their performance in the context of a real pig breeding dataset. We found that family-specific or sire-specific arrays could increase imputation accuracy by 0.11 at one marker per chromosome, by 0.027 at 25 markers per chromosome and by 0.007 at 100 markers per chromosome.

**Conclusions:**

These results suggest that there may be room to use family-specific genotyping for very-low-density arrays particularly if a given sire or sire-dam pairing have a large number of offspring.

## Background

In this paper, we evaluate the value of optimizing the markers that are on low-density genotyping arrays for pedigree-based imputation. Over the past decade, the use of genomic information in livestock breeding has increased substantially and has led to higher accuracy of selection, particularly on traits with a low heritability [1], decreased generational interval for some species [2], and increased rate of genetic gain [3]. Many of these gains have been made possible due to the use of low-cost genotypes obtained through genotype imputation. In the context of animal or plant breeding programs, genotype imputation allows most of the individuals in the population to be genotyped with a low-cost and low-density genotype array, while only a small number of individuals (e.g., the sires and top dams) are genotyped with a high-density array. The markers on the low-density array are used to identify shared haplotypes between low-density and high-density genotyped individuals. The shared haplotype segments are then used to fill-in missing genotypes [3].

High imputation accuracy is key for maximizing the rate of genetic gain in a population; low imputation accuracy decreases genomic prediction accuracy, which in turn decreases the response to selection [4]. One of the primary factors that influences imputation accuracy is the total number of markers on a low-density genotyping array. If there are too few markers, then it may be challenging to identify correctly the shared haplotypes between low-density and high-density genotyped individuals. Having more markers increases the specificity of detecting shared haplotypes, but also increases the cost of genotyping, which potentially limits the total number of individuals genotyped. An alternative way to increase accuracy is to keep the total number of markers constant, but to choose the markers to be as informative as possible [5–7].

Past work on population-based imputation has shown that selecting markers that have a high minor allele frequency, are evenly spaced throughout the chromosome [7], or co-vary strongly with other markers can improve imputation accuracy [5]. These three factors allow a population-based imputation method to distinguish between high-density reference haplotypes and find the specific reference haplotype that the low-density individual carries. For example, markers with a high minor allele frequency are likely to segregate between haplotypes, thus allowing similar haplotypes to be distinguished. In contrast, markers with a low minor allele frequency may be fixed in most of the haplotypes in the population and thus provide limited power to discriminate between haplotypes.

The gains for optimizing the distribution of markers across a chromosome can be substantial, particularly at lower marker densities. Aliloo et al. [5] reported a 0.1 increase in imputation accuracy by using an optimized set of 3757 markers compared to a random set. The relative increase was much smaller at higher marker densities, e.g., the increase was only 0.02 at 11,773 markers genome-wide, which suggests that as the number of markers on a low-density panel increases, the value of any marker selection strategy decreases. It is well known that pedigree-based imputation algorithms have higher imputation accuracies than population-based imputation algorithms at low marker densities [8]. Therefore, the gains by using an optimized marker selection strategy may also occur at relatively lower marker densities.

**Fig. 1:**
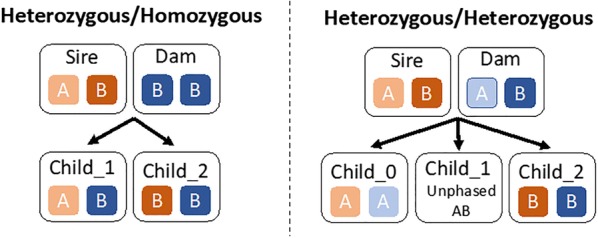
A graphical representation of informative markers for pedigree-based imputation

Unlike in the context of population-based imputation where informative markers depend on the distribution of haplotypes in the population, in pedigree-based imputation, informative markers only depend on the four parental haplotypes. In particular, informative markers are markers that distinguish between each set of parental haplotypes. If the parents have high-density genotypes (potentially by being imputed themselves) and are phased, then the informative markers will be the markers that are heterozygous in the parents. To illustrate this, suppose there is a biallelic marker for which both parents are genotyped and phased. If the sire is *AB* and the dam is *BB*, then the marker is informative for distinguishing sire haplotypes. The resulting offspring will be either *AB* or *BB*. If the offspring is *AB*, we know that it inherited the *A* allele from the sire and since the sire is phased, we know which haplotype the offspring inherited at that marker. Alternatively, if the offspring is *BB*, we know it inherited the *B* allele from the sire and the corresponding haplotype. If both parents are heterozygous at a marker (*AB* and *AB*), then the marker will be informative for both parents in half of the cases, i.e., when the offspring is either *AA* or *BB*. If the offspring is *AB*, the marker will not be informative, since we cannot determine the parent of origin for each allele. We illustrate these conditions in Fig. 1.

The fact that marker informativeness depends only on the genotypes of the parents broadens the options for marker selection strategies beyond optimizing the minor allele frequency and marker spacing only. This is particularly the case if multiple SNP arrays can be developed at low cost. For example, it is reasonable to expect gains in imputation accuracy if the low-density marker array is updated on a yearly basis to track the changing minor allele frequency of the sires and dams in the population, or if multiple panels can be developed to track the allele frequencies in specific subpopulations (e.g., at a given farm or for a given breed/line). At the extreme, we could consider constructing family-specific genotyping panels.

In this study, our aim was to evaluate different marker selection strategies in the context of pedigree-based imputation algorithms and to quantify the extent to which imputation accuracy could be increased by using an optimized marker selection strategy, and determine the marker densities required for those accuracy gains to be realized. To do this, we focused on three methods for creating a single SNP array for a population, and four family-specific methods. These methods place an upper and lower bound on the performance of any marker selection strategy because they represent the extremes: either a single array that was optimized for the entire population, which represents what is currently done in practice, or a family-specific array, which represents the best possible imputation accuracy that can be obtained for a given marker density (i.e., because every marker is informative). Alternative strategies, which are likely to be more economically viable, will fall between the two extremes.

## Methods

### Genetic data

In this study, we used genotypes for 1000 focal individuals and their ancestors from a large commercial pig breeding program. The focal individuals were selected such that they were genotyped on a high-density array (~ 50k markers across the 18 pig autosomes), and had five generations of genotyped ancestors. Some of the ancestors were genotyped at a lower density, but all individuals were imputed to high-density. Although the focal individuals were not chosen to be explicitly related, we found that the focal individuals were the offspring of 50 unique sires and 300 unique dams. In total, we extracted the genotypes for 2405 animals (1000 focal individuals and 1405 ancestors). Then, we performed several simulations in which the genotypes of the focal individuals were masked according to a low-density marker selection strategy (explained below) and imputed based on the un-masked genotypes of the ancestors using AlphaPeel. AlphaPeel is a pedigree-based imputation method based on multi-locus peeling [9]. We ran AlphaPeel by using its default parameters.

### Marker selection strategies

We evaluated two sets of marker selection strategies in which the markers on the low-density array were optimized either for the whole population, or for a specific family. For all methods, we split the chromosome into $k$ bins, where $k$ is the number of low-density markers, and used a marker selection strategy to select a marker from each bin. For each marker selection strategy, we varied the number of low-density markers per chromosome between 1 and 700 in 16 increments, using 1, 2, 3, 5, 10, 15, 25, 50, 100, 150, 200, 300, 400, 500, 600, or 700 markers.

We evaluated three population-based marker selection strategies. We selected either the middle marker from each bin (*midpoint*), the marker in the bin that had the highest minor allele frequency (*maf*), or the marker that was both central and had a high minor allele frequency (*combined*). The centrality combined with high minor allele frequency was based on the method of Wu et al. [7]. For each marker, we calculated a score:

$$score_{i} = - \left( {1 - d_{i} } \right)\left( {p_{i} \log_{2} \left( {p_{i} } \right) + \left( {1 - p_{i} } \right)\log_{2} \left( {1 - p_{i} } \right)} \right),$$

where $d_{i}$ is the distance (in number of markers) between the marker and the centre of the bin, and $p_{i}$ is the minor allele frequency for marker $i$. The term ($1 - d_{i}$) gives a higher weight to markers that are close to the centre of the bin. The term $\left( {p_{i} \log_{2} \left( {p_{i} } \right) + \left( {1 - p_{i} } \right)\log_{2} \left( {1 - p_{i} } \right)} \right)$ is the Shannon information content of the marker based on the minor allele frequency and is highest for markers with a minor allele frequency close to 0.5 [7]. Unlike Wu et al. [7], we did not perform a global optimization of the location of each marker, but instead selected the marker for each bin independently.

Previous work showed that selecting two markers from the first and last bins on the chromosome can improve imputation accuracy because of the higher than normal recombination rate at the ends of the chromosome [6]. Due to the small number of markers used in our study (in some cases, only one marker was used), we selected only one marker from each bin, even for the first and last bins.

We evaluated four family-specific marker selection strategies. We selected the marker that was closest to the centre of the bin and was heterozygous in both parents (*Het*/*Het*), or heterozygous in one parent and homozygous in the other (*Het*/*Hom*), or heterozygous in at least one parent (*Het*/*Any*), or heterozygous in the sire (*Het*/*Sire*). In the Het/Hom condition, we used $\frac{k}{2}$ bins and separately selected markers in each bin that were informative for the sire or the dam (if the number of markers was odd, the sire received $\frac{k + 1}{2}$ bins, and the dam received $\frac{k - 1}{2}$ bins). If a bin did not contain an acceptable marker for the family-specific strategy, we used the *combined* population strategy to select the marker for that bin. This occurred more frequently when the number of low-density markers was large and the number of markers per bin was small, and in the *Het*/*Het* and *Het*/*Hom* conditions that had the smallest pools of acceptable markers.

For all family-specific strategies, we restricted the pool of potential markers to markers that were genotyped in the real dataset (i.e., not missing) in the offspring. In addition, we required that the markers be genotyped for the sire in the *Het*/*Sire* condition, for at least one parent in the *Het*/*Any* condition, and for both the sire and the dam in the *Het*/*Het* and *Het*/*Hom* conditions. This generally produced a small reduction in the number of markers since most offspring were pre-selected such that they were genotyped at high-density, and most sires and many dams were genotyped at high-density. Because the pattern of missing data differed in each individual, we generated family-specific arrays separately for each individual, even when individuals came from the same full-sib family.

### Measurement of imputation accuracy

Imputation accuracy was measured as the correlation between an individual’s imputed genotype and their true genotype, corrected for their parent average genotype:

1 $$accuracy = cor(G_{imputed} - G_{parent\_average} ,G_{true} - G_{parent\_average}).$$

This measure of imputation accuracy is designed specifically for pedigree-based imputation. It is equal to 0 if no genotype information is available on a focal individual (resulting in the individual being imputed as the parent average genotype), and to 1 if the individual is imputed perfectly. The goal of this metric is to assess the accuracy of imputing within-family (Mendelian sampling) genotype variation. Based on simulations, we showed a close relationship between this measure of imputation accuracy and the accuracy of the breeding value estimates (see “Appendix”). In addition, this measure does not rely on using the population minor allele frequency (as opposed to correcting for major allele frequency, as in [4], which may not be representative of the allele frequencies in specific families. In cases for which the genotypes of the parents were missing in the real dataset, we used the imputed values from AlphaPeel to calculate the parent average genotype. This was done primarily to fill-in spontaneous missing genotypes, and to impute dams that were genotyped at a lower density.

Imputation accuracies were calculated separately for each chromosome and then averaged across all 18 autosomes.

## Results

**Fig. 2:**
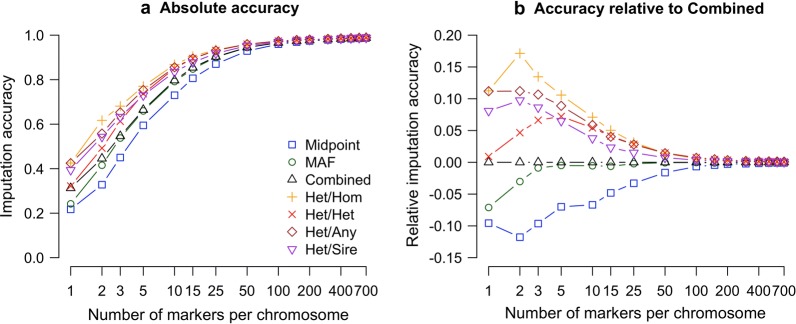
Imputation accuracy as a function of the number of markers per chromosome and the marker selection strategy. **a** Provides the absolute imputation accuracy (measured as correlation between the true and imputed genotypes of an individuals corrected for parent average genotype), while **b** provides comparison relative to the *combined* strategy

Figure 2 presents the performance of using either a population-based strategy or a family-specific strategy, for both the (a) absolute imputation accuracy or (b) imputation accuracy relative to the *combined* population strategy. We found that the *combined* strategy was the highest performing population strategy, followed by the *maf* strategy, and then by the *midpoint* strategy. The difference between the *combined* strategy and the *maf* strategy was less than 0.001 at above 25 markers per chromosome. We found that all of the family-specific strategies outperformed the *combined* strategy. In particular, we found that the *Het*/*Hom* strategy was the highest performing strategy, followed by the *Het*/*Any* strategy, and the *Het*/*Het* strategy. The *Het*/*Sire* strategy performed better than the *Het*/*Het* strategy with less than five markers, but was worse with five or more markers.

The *combined* strategy gave high imputation accuracies across a range of marker densities. Imputation accuracy was equal to 0.312 at one marker per chromosome, 0.796 at 10 markers per chromosome, 0.903 at 25 markers per chromosome, 0.945 at 50 markers per chromosome, and 0.985 at 500 markers per chromosome.

Using a family-specific strategy increased imputation accuracy. When the *Het*/*Any* strategy was used, we obtained an accuracy of 0.424 at one marker per chromosome, 0.855 at 10 markers per chromosome, 0.931 at 25 markers per chromosome, 0.959 at 50 markers per chromosome, and 0.986 at 500 markers per chromosome. The performance of the other family-specific strategies was similar. The increase in imputation accuracy of the family-specific strategies over the *combined* strategy was highest at low marker densities (e.g., 0.11 at one marker per chromosome), and decreased as marker density increased (e.g., 0.001 at 500 markers per chromosome).

**Fig. 3:**
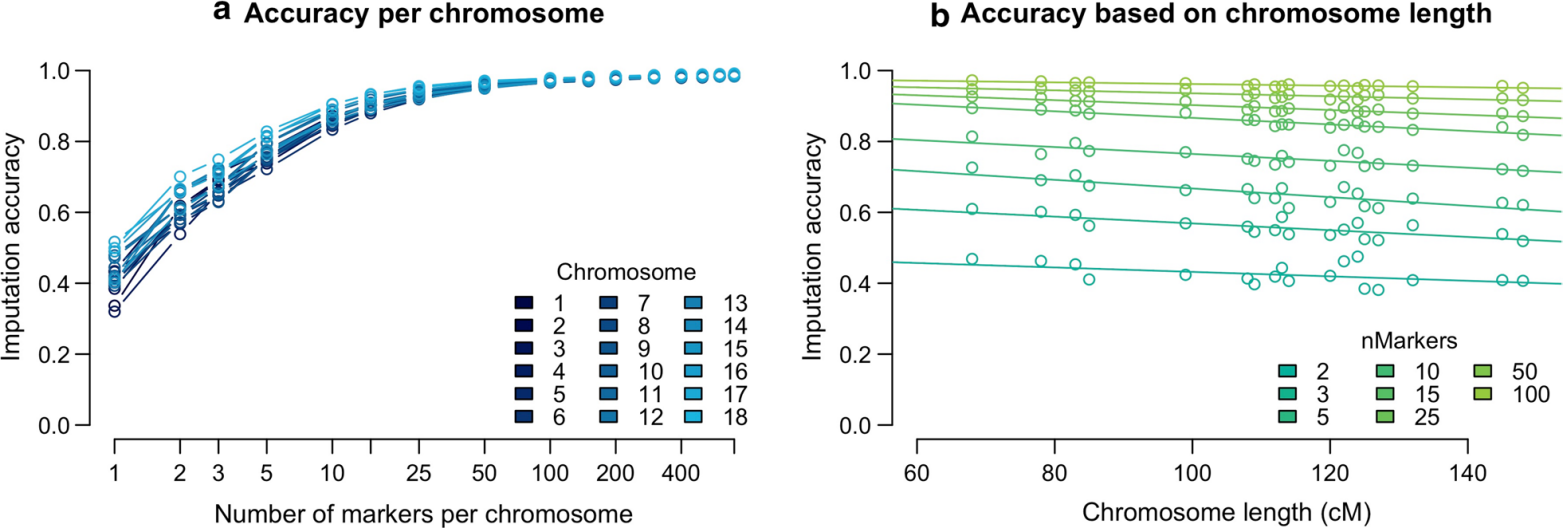
Imputation accuracy by **a** chromosome and **b** chromosome length. In both panels the Het/Any strategy was used to select the markers on the low-density arrays

In Fig. 3a, the imputation accuracy obtained with the *Het*/*Any* strategy is plotted by chromosome and in Fig. 3b by chromosome length. We found that imputation accuracy decreased as the chromosome length increased, but that this difference was small even for large chromosomes. To quantify these differences in imputation accuracy, we used a linear model to measure the effect of the number of markers and chromosome length (in cM) on accuracy. Chromosome lengths were drawn from [10]. The linear model treated chromosome length as a linear covariate and the number of markers as a categorical variable to account for the non-linear effect that number of markers has on accuracy. We found a significant effect of chromosome length on accuracy (regression coefficients decreased from a 0.0012 loss of accuracy per cM at two markers per chromosome to a 0.0001 loss of accuracy per cM at 100 markers per chromosome, p < 0.001) and on the interaction between the number of markers and chromosome length (p < 0.001).

## Discussion

In this paper, we evaluate the performance of using family-specific low-density marker selection strategies to increase the accuracy of pedigree-based imputation. We show that using parental genotype information to select markers on a low-density genotype array increased imputation accuracy, with the largest gains occurring at very low marker densities (between a 0.11 and 0.05 increase in accuracy for between 1 and 25 markers per chromosome), and with more limited gains occurring at higher marker densities (less than a 0.01 increase in accuracy at more than 100 markers per chromosome). In addition, we quantified the influence of chromosome length on imputation accuracy and found that increasing chromosome length had a near-linear impact on imputation accuracy when the number of informative markers per chromosome was kept constant. In the remainder of the discussion, we will highlight the performance of each family-specific marker selection strategy, compare our results to previous studies on the optimization of the design of low-density arrays for population-based imputation, and discuss the commercial viability of using family-specific genotype arrays.

### Performance of family-specific marker selection strategies

Our findings show that selecting the markers on a low-density genotype array based on parental information increased accuracy in all cases compared to using the same set of markers for every individual in the population. We evaluated four marker selection strategies and found that selecting markers that were heterozygous in one parent and homozygous in the other (*Het*/*Hom*, Fig. 1a) yielded the highest imputation accuracies. Selecting markers that were heterozygous in both parents (*Het*/*Het*, Fig. 1b) resulted in much lower imputation accuracies than the *Het*/*Hom* strategy, which is likely due to markers in the *Het*/*Het* condition not being informative if the offspring are heterozygous (Fig. 1b).

In addition to the strategies presented in Fig. 1, we also investigated two hybrid strategies. The first one selected markers that were heterozygous in either parent (*Het*/*Any*), and the second selected markers that were heterozygous in the sire (*Het*/*Sire*). We found that the performance of the *Het*/*Any* strategy was in between that of the *Het*/*Hom* and *Het*/*Het* strategies, which reflects the fact that the chosen markers were split between those that were heterozygous in one parent and homozygous in the other, and those that were heterozygous in both parents. We found that the *Het*/*Sire* condition performed well with a few markers per chromosome, but that the gain in imputation accuracy declined more rapidly compared to the alternative strategies. This is likely due to the *Het*/*Sire* strategy placing most of its weight on finding markers that are informative for the sire, and thus resulting in few markers being informative for the dam. All the same, the *Het*/*Sire* strategy outperformed all of the population-based strategies tested, making it a potentially useful strategy when a single sire produces a large number of offspring.

We focused on the family-specific marker selection strategies because they concentrate all their genotyping effort on informative loci, and thus these strategies provide an upper bound on the imputation accuracy for any alternative strategy for a fixed marker density. This is particularly important given that the development of a family-specific array will not be economically viable in many cases, particularly if the number of individuals per full-sib family is small, and the cost for developing a new array is high. However, there are alternative strategies that might be more economical, e.g., developing a new low-density array for each generation or each year based on the minor allele frequencies of sires and dams in the population, or using separate arrays for different breeds or lines. These arrays may produce a larger number of informative markers than a population-based array, but less than a family-specific array. Because of this, we expect these arrays to be useful if less than 100 markers per chromosome are genotyped. At higher densities, these strategies will likely perform similarly to using a single low-density array for the entire population.

### Comparison to population-based imputation

Our results align closely with those of previous reports on the optimization of low-density genotyping arrays for population-based imputation. Similar to both Aliloo et al. [5] and Wu et al. [7], we found that the gains in imputation accuracy for an optimized array were highest at low-marker densities and decreased at higher densities. We were also able to replicate the primary finding of Wu et al. [7], i.e. that simultaneously optimizing the low-density markers for both a high minor allele frequency and even spacing improved imputation accuracy particularly at low densities.

One of the primary goals of our work was to quantify at what marker densities it was advantageous to optimize the design of low-density arrays. Based on the results of Aliloo et al. [5], we expected that the performance of any specific marker selection strategy would depend on the number of low-density markers (with higher gains available at lower marker densities). Given that imputation accuracy tends to be higher with pedigree-based imputation compared to population-based imputation [8], we expected that the gains in imputation accuracy for pedigree-based imputation would occur at lower densities than for population-based imputation, which was the case. As an example, Aliloo et al. [5] reported a 0.10 gain in imputation accuracy using an optimized marker selection strategy at ~ 125 markers per chromosome. In contrast, the gain at 100 markers per chromosome was less than 0.01 between using a family-specific marker selection strategy or using the midpoint strategy. Larger gains in imputation accuracy occurred at much lower densities (particularly between 1 and 25 markers per chromosome).

### Commercial feasibility of family-based imputation

The primary question of using family-specific genotype arrays revolves around the cost and the complexity of deploying such arrays in the context of a genetic improvement program. There are two primary issues: (1) in order for a family-specific array to be beneficial, the density of the array needs to be low; and (2) the use of a family-specific array may require the construction of a large number of arrays, which may be prohibitively expensive. We discuss both issues in more detail below.

On the question of marker densities, we showed that in order for a family-specific genotype array to be beneficial, the underlying marker density has to be much lower than that traditionally used in an animal improvement program (< 25 markers per chromosome), and will result in lower absolute values of imputation accuracy than a traditional low or medium density array. This limits the use of family-specific arrays when it is acceptable to have imperfect genetic information, i.e., when the accuracy of selection can be low, or when selection decisions are not directly made on the genotyped individual. Such situations might include genotyping individuals in a non-nucleus environment to establish a flow of phenotypic information to individuals in the nucleus or performing genetic improvement in breeding programs for which very low-density arrays are used to genotype a very large number of offspring. This might have potential in aquaculture [11, 12] and crop breeding [13, 14].

Regarding the question about the number of arrays, because family-specific genotype arrays depend on the genotypes of both the sire and the dam, the number of different arrays that are necessary to genotype individuals in the population may be large. This will be particularly the case when a single dam has a limited number of offspring (most notably in cattle and small ruminants, but also in pigs). In these cases, it may be possible to reduce the number of arrays needed by using a sire-specific genotype array. Alternatively, there may be situations where a single sire-dam pair may produce a large number of offspring as is the case in aquaculture and crop breeding, where a single array for multiple individuals could be developed, or where a more flexible genotyping method could be deployed [15].

## Conclusions

Overall, in this paper we evaluate the utility of family information to select markers on a low-density array. Although we find minimal gains at the densities that are currently used in modern breeding programs (over 100 markers per chromosome), the increases in accuracy at very low marker densities (between 1 and 25 markers per chromosome) were high. These results suggest that, for traditional animal breeding programs, the use of multiple low-density panels is likely not beneficial, and that there is little gain in optimizing the markers selected for the panel, beyond selecting markers that have a high minor allele frequency and are evenly spaced across the chromosome. However, for breeding programs for which the number of individuals in a full-sib family is large such as for some plants, aquaculture or insect species, and for which individuals would be genotyped at very low densities, there may be value in carefully selecting which markers to place on a low-density array, and potential advantage in constructing family-specific low-density arrays.

## Figures and Tables

**Fig. 4:**
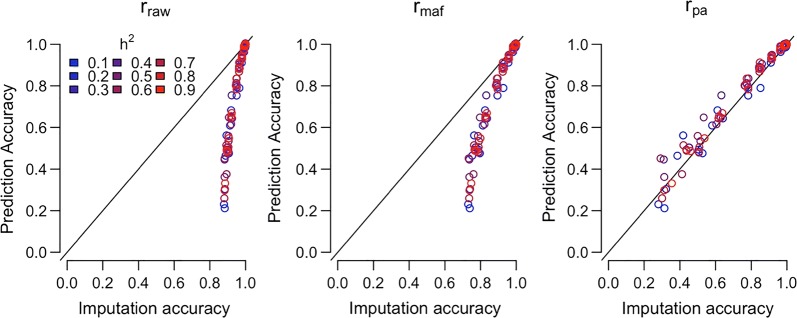
Relationship between relative prediction accuracy and imputation accuracy and when imputation accuracy is either measured as the raw correlation between individual’s imputed genotypes and their true genotypes (r_raw_), or when it is corrected for twice the population allele frequency (r_maf_), or twice the family allele frequency (parent average genotype) (r_pa_)

## Data Availability

The base dataset used in this study cannot be made available due to commercial considerations. However, a copy of the simulation pipeline using a purely simulated dataset is available from the authors upon request.

## Appendix

In the animal breeding literature, there are many measures of imputation accuracy that differ in subtle ways, which makes it difficult to know which metric to use and complicates comparisons between studies. Our ultimate motivation is to use imputation in the context of genomic selection. Thus, we carried out a small simulation study to assess the relationship between three measures of imputation accuracy and the accuracy of genomic prediction using the imputed genotypes.

### Population

In this small study, we simulated five generations of an animal breeding program. We assumed that the first four generations were phenotyped and genotyped at high-density. We assumed that the fifth generation was not phenotyped and was genotyped at low-density. Our goal was to impute the genotypes in the fifth generation and predict the breeding values for those individuals.

We assumed that each generation consisted of 1000 individuals, which were evenly split between males and females. The next generation was created by randomly mating the 50 males and 500 females with the highest true breeding value. We assumed that they were genotyped with 50,000 markers that were evenly spaced across 20 chromosomes of 100 cM. The initial haplotypes in the population were simulated by using a coalescent simulator, MaCS, and ancestral population parameters derived for cattle (as in [9]). The remainder of the simulation was carried out using AlphaSimR (Gaynor et al., personal communications).

We simulated breeding values and phenotypes for a single trait for each individual in the population. To do this, we assumed that all 50,000 markers on the high-density array were quantitative trait loci with additive and normally distributed effects. We varied the heritability of each trait between 0.1 and 0.9 in increments of 0.1.

### Genotypes and imputation

We assumed that high-density genotypes were available on the first four generations, and low-density genotypes were available on the final fifth generation. The low-density genotypes were chosen such that they were evenly spaced across the chromosome, similar to the *midpoint* condition in the main study. Then, we used AlphaPeel to impute the genotypes of each individual in the population. We varied the number of low-density markers per chromosome between 1 and 700 in 16 increments, using 1, 2, 3, 5, 10, 15, 25, 50, 100, 150, 200, 300, 400, 500, 600, or 700 markers.

Imputation accuracy was assessed either by (a) the correlation between an individual’s imputed genotype and their true genotype (*r*_*raw*_), (b) the correlation between an individual’s imputed genotype and their true genotype corrected for twice the population major allele frequency (*r*_*maf*_ as in [4]), or (c) the correlation between an individual’s imputed genotype and their true genotype corrected for twice the family major allele frequency, i.e., the parent average genotype (*r*_*pa*_, as in Eq. 1 in the main study).

### Breeding value prediction

We predicted the breeding values of the last generation using a ridge regression model [16, 17]. The training population was assumed to consist of all the individuals in the first four generations. We predicted the breeding values of the final generation using either the imputed genotypes or the true genotypes. Prediction accuracy was assessed by evaluating the accuracy of the Mendelian sampling term, that is, the correlation between an individual’s true and estimated genetic deviation from the mean parental breeding value. The relative prediction accuracy was calculated as the ratio of the imputation accuracy using imputed genotypes to the prediction accuracy using the true genotypes:

$$acc_{rel} = acc_{imputed} /acc_{true}$$

The performance of a given measure of imputation accuracy was assessed by evaluating the correlation and the regression coefficient between each measure of imputation accuracy and relative prediction accuracy. If a measure of imputation accuracy is a good proxy for the resulting prediction accuracy, we expect that both the correlation coefficient and the regression coefficients will be close to 1. If the regression coefficient is greater than 1, this suggests that the measure of imputation accuracy overestimates the resulting prediction accuracy. If the regression coefficient is less than 1, this suggests that the measure of imputation accuracy underestimates the resulting prediction accuracy.

### Results and discussion

Figure 4 shows that all three measures of imputation accuracy correlate strongly with the relative prediction accuracy, (r_raw_, r = 0.979, p < 0.001; r_maf_, r = 0.981 p < 0.001; *r*_*pa*_, r = 0.989 p < 0.001), but that r_pa_ (b = 0.958) has the regression coefficient closest to 1, followed by r_maf_ (b = 2.345) and r_raw_ (b = 5.161).

These results suggest that using either the raw individual-wise correlation or the individual-wise correlation corrected for twice the population allele frequency will overestimate the usefulness of using the imputed genotypes for predicting the Mendelian sampling term. In contrast, correcting for twice the family allele frequency (parent average genotype) will provide a less biased estimate of the relative information loss with imputed data. These results differ from those reported by Calus et al. [4], probably because here, we focus on pedigree-based imputation. In the context of pedigree-based imputation, accurate genotypes can be constructed by setting the genotypes of the offspring equal to the parent average genotype. This will provide high accuracy when using the raw correlation or when correcting for twice the population allele frequency, but will not allow a breeder to distinguish between full-sibs who will be all imputed to the same value (i.e., the Mendelian sampling terms). In contrast, correcting for twice the family allele frequency (parent average genotype) allows a more accurate assessment of the ability to distinguish between full-sibs, and the overall utility of the genetic information.
